# Evaluation of batch fraction, corn silage inclusion level, and mixing duration on long particle distribution of finishing diets for beef cattle

**DOI:** 10.12688/f1000research.25981.1

**Published:** 2020-09-02

**Authors:** Elizabeth M. Buckhaus, Dathan T. Smerchek, Zachary K. Smith

**Affiliations:** 1 Department of Animal Science, South Dakota State University, Brookings, SD, 57007, USA

**Keywords:** corn silage, finishing diet, mixing duration, particle size

## Abstract

**Background: ** Differing fractions of a batch of feed, differing ingredient characteristics, and inadequate mix time can lead to non-uniformity within a mix of feed.

**Methods: **The experiment was designed as a 5 x 2 x 2 factorial arrangement with seven replications per simple treatment mean. Factors included: 1) batch fraction (BF; n = 5); 2) corn silage inclusion level (CSLVL; n = 2) 15% or 30% inclusion (dry matter basis); and 3) mixing duration (DR; n = 2) of 20 or 25 mixer revolutions. Data were analyzed as a completely randomized design using a binomial approach. The Penn State Particle Separator was used to separate fractions of the total mixed ration (TMR).

**Results: **No interactions between BF, CSLVL, and DR were detected ( *P *≥ 0.31) for any dependent variables. There was an increase ( *P *= 0.01) in retention on the 19 mm sieve from the first BF compared to the last BF. CSLVL altered ( *P *= 0.01) retention on the 19 mm sieve. Increasing DR from 20 to 25 revolutions had no appreciable influence ( *P *= 0.23) on particles greater than 19 mm.  CSLVL ( *P *= 0.01) and DR ( *P *= 0.01) altered particle retention on the 8 mm sieve. BF ( *P *= 0.01), CSLVL ( *P *= 0.01), and DR ( *P* = 0.02), influenced particle retention on the 4 mm sieve. CSLVL impacted ( *P *≤ 0.01) particles remaining in the bottom pan and particles greater than 4 mm. BF ( *P *= 0.01) and CSLVL ( *P* = 0.01) altered particles greater than 8 mm.

**Conclusions:** These data indicate that BF and CSLVL fed alters particle size distribution that in turn could alter dry matter intake, dietary net energy content, and influence daily gain. Mixing DR had no appreciable influence on particle size distribution of the TMR.

## Introduction

Varying feed ingredient properties such as particle size, shape, density, hygroscopocity, static charge, and adhesiveness can influence how a beef cattle diet mixes prior to feeding. Differing fractions of a batch of feed, differing ingredient characteristics, and mix time can also lead to non-uniformity within a specific mix of feed. 

Blom *et al.* (2020) demonstrated that as the mixer unloads, there is a linear increase in the proportion of long particles fed that results in greater intake, poorer gain, and reduced gain to feed (average daily gain/dry matter intake) in steers during the feedlot receiving phase. Smerchek *et al.* (2020) demonstrated that as particles greater than 4 mm increase, there is a reduction in average daily gain by approximately 0.02 kg for each percentage point increase in particles greater than 4 mm in the diet.

The objective of this research was to determine how batch fraction, diet roughage level, and mixing duration influenced particle distribution in finishing diets for beef cattle. The hypothesis was that batch fraction would influence the particles size distribution, greater corn silage inclusion (roughage level) would alter particle size distribution, and mixing duration would have no influence on particle size distribution of the total mixed ration.

## Methods

### Ethical statement

No Institutional Animal Care and Use Committee approval was obtained for this experiment since no animals were used to generate the data used in the present analysis. The study was conducted at the Ruminant Nutrition Center in Brookings, SD, USA.

### Treatment structure, diet manufacturing, ingredient inclusion order, and total mixed ration separation

The experiment was designed as a 5 × 2 × 2 factorial arrangement with seven replications per simple treatment mean. Factors included: 1) batch fraction (BF; n = 5), where BF 1 was the first 20% of feed unloaded from the mixer and BF 5 was the last 20% of feed unloaded from the mixer; 2) corn silage inclusion level (CSLVL; n = 2) containing (dry matter basis) 15% corn silage or 30% corn silage replacing the corn blend; and 3) mixing duration (DR; n = 2) of 20 or 25 mixer revolutions (5 revolutions·minute ^-1^) prior to unloading. A 2.35 m ^3^ horizontal mixer (Roto-Mix; Dodge City, KS) was used to manufacture all diets. Diets contained corn silage, a 1:1 ratio of dry-rolled corn:high-moisture corn, a liquid supplement (5% dry matter inclusion), and a meal supplement (7% dry matter inclusion). Ingredients were added into the horizontal mixer in the following sequence: high-moisture corn, dry-rolled corn, liquid supplement, dry supplement, and finally corn silage.

 The total mixed ration (TMR) samples were separated using the Penn State Particle Separator (PSPS) using the methods described by ( Kononoff *et al.*, 2003). The PSPS had three sieves (19 mm, 8 mm, 4 mm, and pan). The particles retained on the top sieve (19 mm) were considered large, middle sieve (8 mm) were considered medium, and bottom sieve (4 mm) were considered small. Particles less than 4 mm were collected in the pan. Proportions of the TMR on differing sieves was determined on an as-is basis.

### Statistical analysis

Data were analyzed as a completely randomized design appropriate for a 5 × 2 × 2 factorial arrangement of treatments using the GLIMMIX procedure of SAS 9.4 (SAS Inst., Inc., Cary, NC) using a binomial approach. There was a total of seven replications for each simple treatment mean (the combination of each BF, CSLVL, and DR). All data are presented as least squares means and the corresponding standard error of the mean. An α of 0.05 was used to determine significance.

## Results

**Figure 1.**
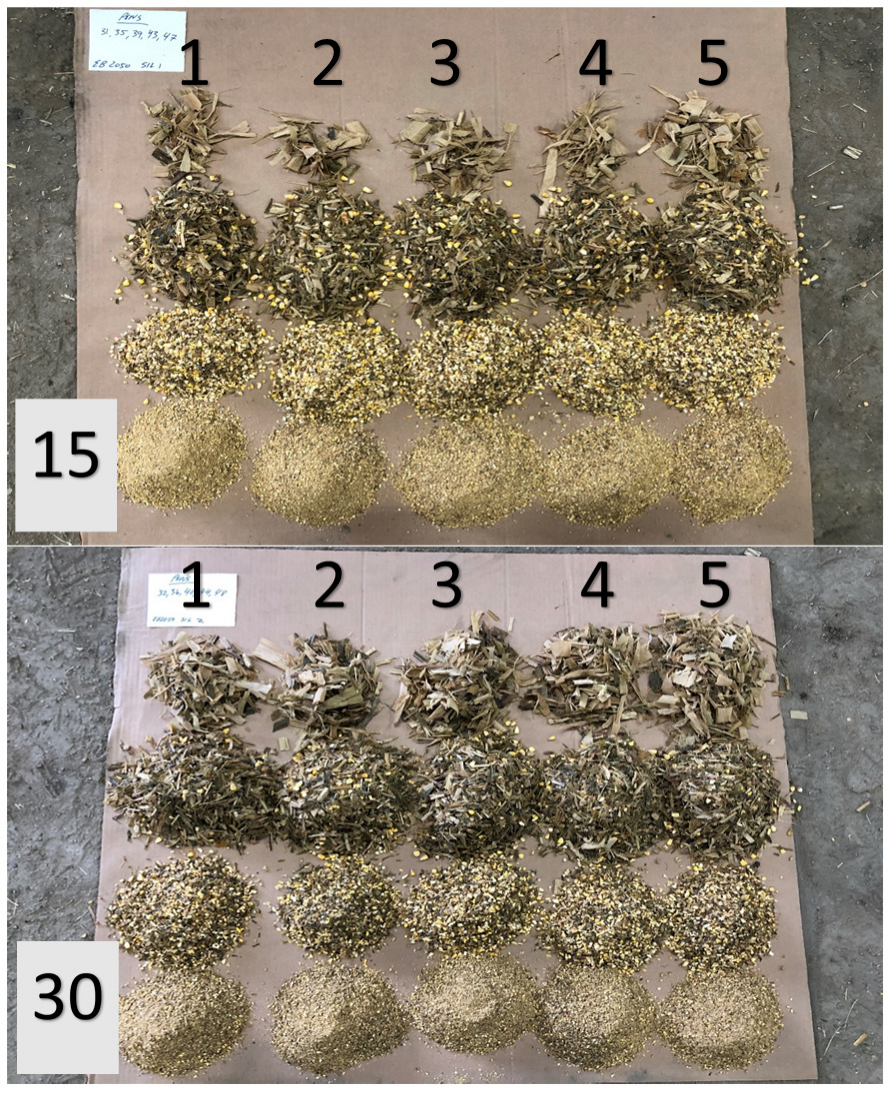
Visual illustration of the batch fraction (1, 2, 3, 4, or 5) and corn silage percentage (dry matter basis) fed (15 or 30) for the diets that were mixed for 20 revolutions.

The effect of BF, CSLVL, and DR on TMR particle size distribution are presented in Table 1 and full results are available as *Underlying data* ( Smith *et al.*, 2020). Visual representation of the TMR’s fed are shown in Figure 1 (20 DR only). No interactions between BF, DIET, and REV were detected ( *P* ≥ 0.31) for any dependent variables. There was a 53.5% increase ( *P* = 0.01) in retention on the 19 mm sieve from the first BF (first 20% of the TMR unloaded from the mixer) compared to the last BF (last 20% of the TMR unloaded from the mixer). The 15 CSLVL diet had a 71.3% decrease ( *P* = 0.01) in retention on the 19 mm sieve compared to the 30 CSLVL diet. Increasing DR from 20 to 25 revolutions had no appreciable influence ( *P* = 0.23) on particles greater than 19 mm. CSLVL ( *P* = 0.01) and DR ( *P* = 0.01) altered particle retention on the 8 mm sieve. BF ( *P* = 0.01), CSLVL ( *P* = 0.01), and DR ( *P* = 0.02) influenced particle retention on 4 mm sieve. CSLVL impacted ( *P* ≤ 0.01) particles remaining in the bottom pan and particle greater than 4 mm. BF ( *P* = 0.01) and CSLVL ( *P* = 0.01) altered particles greater than 8 mm.

**Table 1.** Influence batch fraction (BF), corn silage inclusion level (CSLVL), and mixing duration (DR) on particle size distribution of the total mixed ration (TMR) finishing diet ^1^.

	BF						CSLVL			DR			*P* - values						
Item	1	2	3	4	5	SEM ^2^	15%	30%	SEM	20	25	SEM	BF	CSLVL	DR	BF × CSLVL	BF × DR	CSLVL × DR	BF × CSLVL × DR
Replicates, n	28	28	28	28	28	-	70	70	-	70	70	-	-	-	-	-	-	-	-
TMR, % (as-is basis)																			
Large (≥ 19 mm)	4.0 ^c^	4.0 ^c^	4.4b ^c^	5.2 ^b^	6.2 ^a^	0.34	2.1	7.4	0.22	4.6	5.0	0.22	0.01	0.01	0.23	0.02	0.54	0.39	0.44
Medium (8 to 19 mm)	33.4	33.1	32.9	33.2	33.3	0.32	29.9	36.5	0.20	33.6	32.7	0.20	0.81	0.01	0.01	0.10	0.85	0.41	0.42
Small ( 4 to 8 mm)	29.8 ^a^	29.4 ^ab^	29.0b ^c^	28.4 ^cd^	27.9 ^d^	0.16	31.5	26.3	0.16	28.7	29.2	0.16	0.01	0.01	0.02	0.14	0.59	0.13	0.35
Less than 4 mm	32.8	33.4	33.7	33.2	32.5	0.40	36.5	29.8	0.25	33.1	33.1	0.25	0.26	0.01	0.97	0.34	0.95	0.30	0.31
Greater than 4 mm	67.2	66.6	66.3	66.8	67.5	0.40	63.5	70.2	0.25	66.9	66.9	0.25	0.26	0.01	0.97	0.34	0.95	0.29	0.31
Greater than 8 mm	37.4 ^bc^	37.1 ^c^	37.3 ^bc^	38.4 ^b^	39.5 ^a^	0.40	32.0	43.9	0.25	38.2	37.7	0.25	0.01	0.01	0.13	0.12	0.66	0.93	0.32

^1^Determined according to ( Kononoff *et al.*, 2003). ^2^Standard error of the mean. ^a,b^Means with in a row without a common superscript differ ( *P* ≤ 0.05).

## Conclusions

These results indicate that BF and CSLVL influences particle size distribution of the TMR fed to feedlot cattle. This potentially could alter dry matter intake, dietary net energy content, and influence animal average daily gain, by altering the actual diet fed from what was formulated to be fed. Mixing DR had no appreciable influence on particle size distribution of the TMR, a shorter mixing duration could have a pronounced impact on the distribution of particles in the TMR, however, a shorter mix DR was not investigated in the present experiment. Future experiments should determine what the shortest possible mix duration could be to effectively manufacture finishing diets fed to feedlot cattle.

## Data availability

### Underlying data

Figshare: Evaluation of Batch Fraction, Corn Silage Inclusion Level, and Mixing Duration on Long Particle Distribution of Finishing Diets for Beef Cattle ( Smith *et al.*, 2020). https://doi.org/10.6084/m9.figshare.12841469.v1

Data are available under the terms of the Creative Commons Attribution 4.0 International license (CC-BY 4.0).
