# Supplementary material for: Evaluation of batch fraction, corn silage inclusion level, and mixing duration on long particle distribution of finishing diets for beef cattle
Source: F1000Res. 2020 Sep 2;9:1085. [Version 1] doi: 10.12688/f1000research.25981.1 (PMC8063516; doi:10.12688/f1000research.25981.1)
Supplement: Peer review history

# Peer review: Reviewer response for version 1

**DOI:** 10.5256/f1000research.28673.r81344

**Published:** 2021-04-19

**Authors:** Ahmed M. Abd El Tawab

**Affiliations:** 1 Dairy Science Department, National Research Centre, Dokki, Giza, Egypt

1. Novelty Study and Clear the aims and Hypotheses: The particle size of feed plays an important role in animal feed strategies and consequence on productive performance and average daily weight gain. The author has studied to determine how batch fraction, diet roughage level, and mixing duration influenced particle distribution in finishing diets for beef cattle.
2. The title should be changed to Evaluation of batch fraction, corn silage inclusion level, and mixing duration on particles size distribution of finishing diets for beef cattle.
3. The keywords should be included "beef cattle".
4. The introduction should include information about the digestibility.
5.  The materials and methods should be added experimental animal paragraph.
6. Table and figure are good.
7. Results are good. But the Author should add animal performance table beside the finding table.  
8. Conclusion is good.

Is the work clearly and accurately presented and does it cite the current literature?

Yes

If applicable, is the statistical analysis and its interpretation appropriate?

I cannot comment. A qualified statistician is required.

Are all the source data underlying the results available to ensure full reproducibility?

Yes

Is the study design appropriate and is the work technically sound?

Yes

Are the conclusions drawn adequately supported by the results?

Yes

Are sufficient details of methods and analysis provided to allow replication by others?

Yes

Reviewer Expertise:

I am an associate research professor, my scope is ruminants nutrition, animal nutrition, animal production, milk production, dairy and beef cattle, feed evaluation, feed technology and rumen simulation.

I confirm that I have read this submission and believe that I have an appropriate level of expertise to confirm that it is of an acceptable scientific standard.

# Peer review: Reviewer response for version 1

**DOI:** 10.5256/f1000research.28673.r82061

**Published:** 2021-04-08

**Authors:** Maghsoud Besharati

**Affiliations:** 1 Department of Animal Science, University of Tabriz, Ahar, Iran

I would like to congratulate authors for the good-quality of the article, the literature reported used to write the paper, and for the clear and appropriate structure. The manuscript is well written, presented and discussed, and understandable to a specialist readership.     

 In general, the organization and the structure of the article are satisfactory and in agreement with the journal instructions for authors. The subject is adequate with the overall journal scope.       

 The work shows a conscientious study in which a very exhaustive discussion of the literature available has been carried out. The introduction provides sufficient background, and the other sections include results clearly presented and analyzed exhaustively.     

 However, to improve the manuscript, some revisions are recommended such as:    

- English language needs to be checked;
- Update the used literature;
- Revise the paper according to journal's instructions.

1. The novelty of the study needs to be highlighted compare to other similar studies.
2. The scientific background of the topic is poor. In "Introduction" and "Discussion", the authors should cite recent references between 2016-2020 from JCR journals (with impact factor) about recent achievements on the subject.
3. Authors should state something about Selenium and Vitamin E. At least producer of the additives should stated.

So, I recommend the acceptance of the paper after revision.

Is the work clearly and accurately presented and does it cite the current literature?

Yes

If applicable, is the statistical analysis and its interpretation appropriate?

Yes

Are all the source data underlying the results available to ensure full reproducibility?

Yes

Is the study design appropriate and is the work technically sound?

Yes

Are the conclusions drawn adequately supported by the results?

Yes

Are sufficient details of methods and analysis provided to allow replication by others?

Yes

Reviewer Expertise:

Animal Nutrition, Ruminant Nutrition, Dairy cattle, Beef

I confirm that I have read this submission and believe that I have an appropriate level of expertise to confirm that it is of an acceptable scientific standard.

# Response

**Published:** 2021-04-15

**Authors:** Zachary Smith

**Affiliations:** South Dakota State University, USA

The authors appreciate the time you took to review our manuscript.

 Best,

 Zachary Smith

 South Dakota State University
